# [^18^F]fluoride Activation and ^18^F-Labelling in Hydrous Conditions—Towards a Microfluidic Synthesis of PET Radiopharmaceuticals

**DOI:** 10.3390/molecules29010147

**Published:** 2023-12-26

**Authors:** Olga Ovdiichuk, Salla Lahdenpohja, Quentin Béen, Laurent Tanguy, Bertrand Kuhnast, Charlotte Collet-Defossez

**Affiliations:** 1 Nancyclotep, Molecular Imaging Platform, 54500 Vandoeuvre-les-Nancy, France; 2 Université Paris Saclay, CEA Inserm, CNRS, BioMaps, 91401 Orsay, France; 3 PMB-Alcen, 13790 Peynier, France; 4 Université de Lorraine, Inserm, IADI, 54000 Nancy, France

**Keywords:** ^18^F-radiolabelling, radiopharmaceuticals, ^18^F-activation, hydrous radiofluorination, microfluidics

## Abstract

^18^F-labelled radiopharmaceuticals are indispensable in positron emission tomography. The critical step in the preparation of ^18^F-labelled tracers is the anhydrous F-18 nucleophilic substitution reaction, which involves [^18^F]F^−^ anions generated in aqueous media by the cyclotron. For this, azeotropic drying by distillation is widely used in standard synthesisers, but microfluidic systems are often not compatible with such a process. To avoid this step, several methods compatible with aqueous media have been developed. We summarised the existing approaches and two of them have been studied in detail. [^18^F]fluoride elution efficiencies have been investigated under different conditions showing high ^18^F-recovery. Finally, a large scope of precursors has been assessed for radiochemical conversion, and these hydrous labelling techniques have shown their potential for tracer production using a microfluidic approach, more particularly compatible with iMiDEV™ cassette volumes.

## 1. Introduction

Positron emission tomography (PET) imaging represents a powerful technique for molecular diagnostic and therapeutic procedures. The development of suitable PET imaging agents that can be readily labelled with positron-emitting radionuclides is essential for the detection, characterisation and staging of diseases. Molecular imaging using ^18^F-radiolabelled small-molecule probes as imaging agents is successfully used in cardiology, neurodegenerative disease, inflammation diseases, bacterial infection detection and oncology [1].

**Figure 1:**
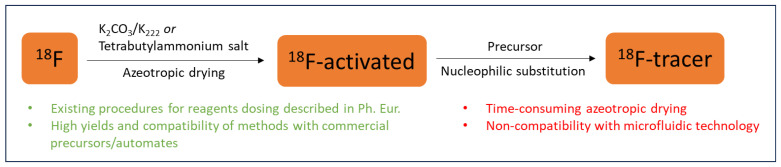
Standard ^18^F-activating methods.

The nucleophilic substitution reaction is the most frequently used strategy for ^18^F-radiolabelling (Figure 1). 

To favour the nucleophilicity of [^18^F]f-anion, the number of hydrogen-bound water molecules should be decreased. Therefore, ^18^F-radiofluorination usually necessitates time-consuming methods, including azeotropic drying prior to labelling to remove water. This evaporation step is commonly implemented on all commercially available synthesisers but remains a limitation to downscale to microfluidic platforms. In the radiochemistry of C-^18^F bonds, a number of studies have described ^18^F-radiofluorination methods without a need for thermal drying while being compatible with a low water content [2,3,4]. These have included: (1) “^18^F-radiofluorination on SPE cartridge” method [5,6]; (2) radiolabelling using polymers either modified with phosphazene bases [7] or loaded with a long quaternary ammonium alkyl chain [8,9]; (3) ^18^F-radiofluorination using strong bases [10,11]; (4) the use of ionic liquids [12,13]; (5) transition metal mediated/catalysed radiofluorination [14,15,16,17,18,19]; (6) cryptate-mediated ^18^F-fluorination [20,21,22,23,24]; (7) radiofluorination using tetraalkylammonium salts (“non-anhydrous, minimally basic (NAMB) approach”) [25,26,27,28,29]; and (8) sulfonyl [^18^F]fluorides as [^18^F]fluoride source [30,31,32]. A summary of these methods is presented in Table 1.

When putting the emphasis on the simplicity of the whole ^18^F-radiofluorination process, hydrous cryptate-mediated ^18^F-fluorination and the use of tetrabutylammonium salts stand out from the drying-free radiofluorination methods. In addition, kryptofix 2.2.2 (K_222_) and tetrabutylammonium salts are widely used in the manufacturing of radiopharmaceuticals, and thus, they are accompanied by a large panel of analytical standardised methods described in the European or US Pharmacopoeia. This is advantageous to establish quality control if a final injection to humans is envisaged. The use of these hydrous elution methods is also supported by earlier results in microfluidic PET tracer production, where small amounts of water (<10%) have been well tolerated [33,34].

In this article, we have exploited these drying-free approaches (Table 1, entries 6 and 7) to evaluate the elution efficiency of fluorine-18 trapped on several types of anion exchangers embedded on the innovative iMiDEV^TM^ microfluidic platform with various amounts of water. We have then assessed the influence of water content on the radiofluorination reaction on seven different precursors of radiotracers and prosthetic reagents. This study represents the first step toward the implementation of full radiosynthesis on the iMiDEV^TM^ microfluidic platform.

## 2. Results

**Figure 2:**
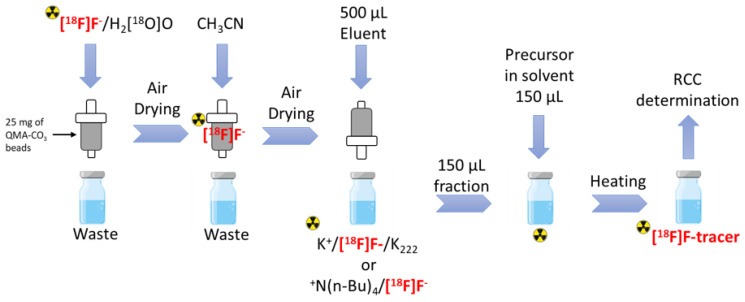
Schematic workflow of the manual [^18^F]fluoride elution in reverse mode and subsequent hydrous labelling.

This study aims to develop a method for [^18^F]fluoride activation at the microfluidic scale suitable for labelling various radiotracers using the iMiDEV^TM^ microfluidic platform. Due to the limited volume that can be used on microfluidic cassettes, an optimisation of the trapping of fluorine-18 on anion exchange beads (AEX), hydrous release and radiofluorination has been performed as described in Figure 2.

### 2.1. [^18^F]fluoride Recovery Studies

Efficient [^18^F]fluoride recovery from strong anion exchange cartridges and its reactivity in the subsequent nucleophilic substitution reaction with versatile precursors is of the utmost importance for developing a robust radiolabelling procedure. Based on a compilation of literature data, we were prompted to evaluate two promising methods on a microvolume scale. The first method reported by Kniess et al. [22] consists of using a kryptofix/K_2_CO_3_ complex (30–60 µmol/15–30 µmol) in a solvent system containing water (2–3%) in acetonitrile (*v*/*v*, 1 mL). The second method developed by Kim et al. [12] uses a low water content (5% *v*/*v*) of the concentrated tetrabutylammonium bicarbonate (40%) in different organic solvents (CH_3_CN, DMF, DMSO, 1 mL). Based on these results, our aim was to optimise the elution protocols for volumes and conditions that could be easily translated onto the microfluidic platform iMiDEV™ where volumes are limited.

The first part of this study aimed to select the best AEX resins among different commercial sources and the most efficient hydrous elution method. The schematic workflow is shown in Figure 2. Three types of commercially available AEX beads from Waters^TM^, Eichrom and S*Pure were used. First, the data obtained from the suppliers on the bead mass in each commercial cartridge was compared to the measured bead mass after opening the cartridge (Table S3 of Supplementary Materials). A discrepancy between the packaging information and the measured data was observed. To standardise the trapping-elution experiments and minimise the bead mass, 25 mg (corresponding to 50 µL bed volume) of each type of resin was filled in an empty SPE cartridge.

Aqueous [^18^F]fluoride (50–200 MBq) was first trapped on the AEX resin, and the ^18^O-enriched water was discarded into the waste vial. The cartridge was then dried with an airflow. Excellent trapping efficiencies were observed for all three types of beads despite the reduced beads quantity. A trapping efficiency of 92 ± 3% (*n* = 3) was measured for beads from Waters^TM^, 95 ± 4% (*n* = 3) from Eichrom and 97 ± 2% (*n* = 3) for those from S*Pure. Residual water was eliminated by passing anhydrous acetonitrile through the cartridges, followed by air drying. The ^18^F-activity loss for the washing step was ≤5% for all experiments. [^18^F]fluoride was gradually eluted in the opposite direction to the trapping using 0.1 mL fractions of the eluent (total elution volume 0.5 mL). Eluents used in the manual tests were 1) kryptofix/K_2_CO_3_ eluent (60 µmol/30 µmol) containing 3% (*v*/*v*) water in CH_3_CN and 2) 5% water (*v*/*v*) TBAB_40%_ solution in acetonitrile or DMSO.

**Figure 3:**
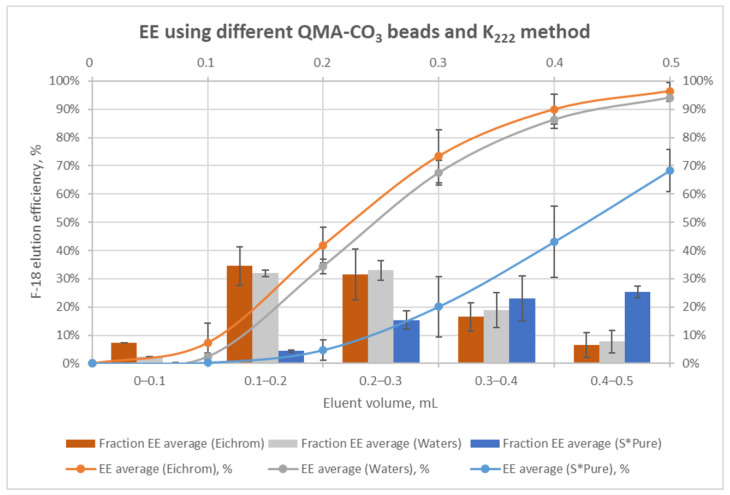
[^18^F]F-elution profile using different types of anion exchange cartridges and the kryptofix-based method.

The activity of the cartridge and the eluate were measured after each elution fraction. Table S4 of Supplementary Materials and Figure 3 show the results of the QMA-CO_3_ elution profile using the kryptofix-based method on different types of QMA-CO_3_ beads. With the K_222_-based method, QMA-CO_3_ beads supplied by Eichrom have shown a faster start of the elution (from the first 0.1 mL). However, overall elution efficiency (EE) was similar to QMA-CO_3_ beads supplied by Waters^TM^. The fractions between 0.1 mL and 0.4 mL (6 bed volumes) contained the highest radioactivity concentrations (around 83% of the total activity). Accordingly, based on the results obtained using AEX resin supplied by S*Pure, the K_222_-based method allowed us to obtain 68 ± 8% elution efficiency with the maximum activity concentrated between 0.2 and 0.5 mL (around 63% of the total activity in 6 bed volumes).

**Figure 4:**
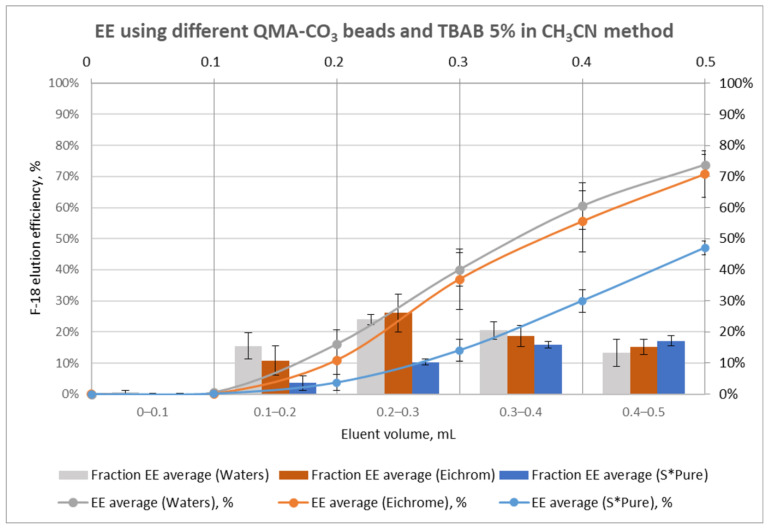
[^18^F]fluoride elution profile using different types of anion exchange cartridges and tetrabutylammonium-based method.

Table S5 of Supplementary Materials and Figure 4 represent the elution efficiencies obtained with 5% H_2_O TBAB_40%_ in acetonitrile. An average recovery of 73.8 ± 3.4% and 70.8 ± 7.4% have been measured for Waters^TM^ and Eichrom AEX beads, respectively. Only half of the trapped activity was eluted under the same conditions when S*Pure beads were used. As for the kryptofix approach, the first 0.1 mL of eluate (2 bed volumes) did not contain any ^18^F-anions.

Significantly lower elution efficiency was observed with 5% water TBAB_40%_ when acetonitrile was replaced by DMSO as a solvent, allowing only 11.5% (*n* = 1) ^18^F-recovery from AEX cartridges. Therefore, all following tests were performed with phase-transfer catalysts (PTC) in acetonitrile solution.

This first study led us to select QMA-CO_3_ beads from the Waters™ and the kryptofix-based hydrous elution method to be transferred to the iMiDEV^TM^ microfluidic platform. In the second part of this study, different elution conditions were evaluated using a microfluidic cassette in which reactor 1 (R1, dedicated to the trapping and concentration of [^18^F]F^−^) was filled with QMA-CO_3_ beads from Waters^TM^. The trapping efficiency of [^18^F]fluoride in QMA-CO_3_ beads using the microfluidic cassette was 99.0 ± 0.9% (*n* = 20). Activity loss during the resin washing step with CH_3_CN was 0.8 ± 1.0% (*n* > 50, decay corrected).

**Figure 5:**
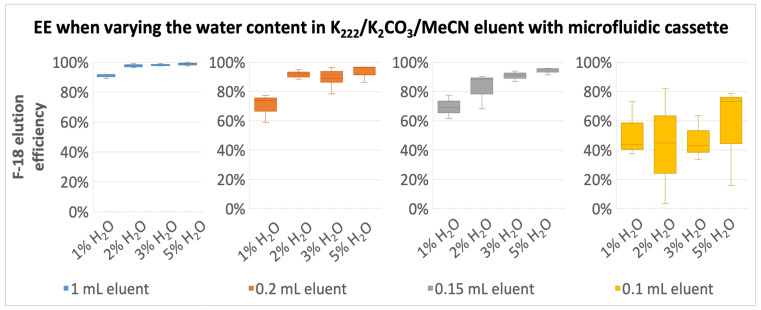
[^18^F]fluoride elution profile using the kryptofix-based elution method in a microfluidic cassette varying the water content and eluent volume.

Preliminary tests with direct trapping and the elution approach gave poor elution efficiencies when using small elution volumes; thus, we chose to continue only with the reversed trapping-elution approach (Figure 2). Elution results using the kryptofix-based method are presented in Figure 5 and Table S6 of Supplementary Materials. To evaluate the importance of water for the elution efficiency, 1% to 5% water-containing elution solutions were studied. Throughout the data, the elution efficiency showed an increasing trend when the water content was increased (Figure 5). With 2% or more water in the eluent, EE was over 97% when ≥20 bed volumes (≥1 mL) of eluent were used. Decreasing the water content to 1% dropped the EE to 90.8 ± 1.2% with ≥20 bed volumes of eluent. When decreasing the eluent volume to 200 µL (4 bed volumes), the EE variance increased, but the EE stayed satisfactory when the water content was at least 2%. With 200 µL of 1% water containing eluent, the EE variance was the highest and EE was only up to 80%. Decreasing the eluent volume to 150 µL (3 bed volumes) gave similar results as 200 µL. Further reduction of the eluent volume to 100 µL (2 bed volumes) had a drastic decreasing effect on EE, and elution was not reproducible with any amount of water as can be seen in Figure 5.

### 2.2. [^18^F]fluoride Labelling Studies

**Figure 6:**
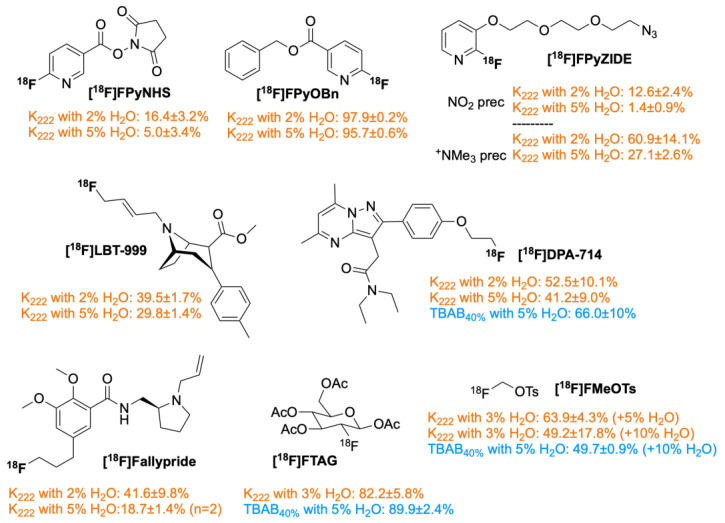
Chemical structures of different radiopharmaceuticals and radiopharmaceutical intermediates were obtained in this study under drying-free nucleophilic ^18^F-fluorination conditions. RCC rates as measured from radio-TLC (*n* = 3).

To investigate the applicability of these two different [^18^F]fluoride activation approaches for the subsequent radiolabelling, the obtained eluates have been tested in manual radiolabelling of different precursors at a microvolume scale. Reaction volumes were adjusted to be compatible with the iMiDEV™ microfluidic platform (volume of the reaction chamber R2 < 290µL). Figure 6 (Table S1 of Supplementary Materials) represents the results of the hydrous nucleophilic ^18^F-fluorination of various radiotracer and prosthetic reagent precursors. Aliphatic and heteroaromatic precursors have been selected for a large validation scope.

[^18^F]FDG, representing the gold standard of PET imaging, was the first selected radiopharmaceutical to be tested. The synthesis of [^18^F]FDG has been widely studied with various synthesisers, from conventional to chip [35]. The intermediate molecule [^18^F]FTAG has been synthesised from only 5 mg of mannose triflate precursor under hydrous conditions with an excellent RCC of 82.2 ± 5.8% and 89.9 ± 2.4% using kryptofix/K_2_CO_3_ (with 3% of water) and TBAB (with 5% of water) activation methods, respectively (Table S1, entries 1, 2 of Supplementary Materials).

Tosylated precursors of [^18^F]fluoromethylcholine ([^18^F]FCH) and [^18^F]DPA-714 underwent radiofluorination using both hydrous approaches. Firstly, the fluorination of methylditosylate was assessed by performing the labelling at 120 °C for 10 min [36]. The intermediate [^18^F]F-Me-OTs was obtained in up to 63.9 ± 4.3% RCC under the kryptofix-based method as evaluated by the radio-TLC (Table S1, entries 3, 4 of Supplementary Materials). The radiofluorination using 5% water TBAB_40%_ in CH_3_CN and 7 mg of precursor in MeCN/H_2_O 8/2 *v*/*v* mixture under the same heating conditions resulted in 49.7 ± 0.9% RCCs. This is similar to the RCC obtained using a kryptofix solution with 20% water content in the precursor vial, suggesting a greater impact of the water content than the F-18 activation method (Table S1, entries 4, 5 of Supplementary Materials). The impact of water on the preparation of this radioactive intermediate has already been reported in the literature. Pascali et al. have also implemented the radiosynthesis of ^18^F-choline on a microfluidic device [36,37,38]. Secondly, radiofluorination of TsO-DPA-714 using 2% or 5% water containing the kryptofix elution solution gave 52.5 ± 10.1% and 41.2 ± 9.0% RCC, respectively, with 10 min reaction at 130 °C in DMSO (based on radio-TLC analysis). Using 5% water TBAB_40%_/CH_3_CN eluent, reaction at 130 °C for 10 min in DMSO resulted in a 66.0 ± 10% RCC into [^18^F]DPA-714 as assessed by radio-HPLC (Table S1, entries 6–8 of Supplementary Materials). Tosylated precursor of [^18^F]fallypride and chlorinated precursor of [^18^F]LBT-999 underwent ^18^F-fluorination using a kryptofix-based aqueous approach (2%/5% H_2_O) and 10 min reaction at 130 °C in DMSO (Table S1, entries 9–12 of Supplementary Materials). Radiolabelling of [^18^F]fallypride resulted in 41.6 ± 9.8% (2% H_2_O) and 18.7 ± 1.4% (*n* = 2, 5% H_2_O) RCC. [^18^F]LBT-999 was produced in 39.5 ± 1.7% (2% H_2_O) and 29.8 ± 1.4% (5% H_2_O) RCC (Figure 6).

To evaluate the compatibility of hydrous ^18^F-fluorination in the preparation of different prosthetic reagents, three different precursors with different leaving groups were labelled using the kryptofix-based aqueous approach (2%/5% H_2_O). [^18^F]FPyNHS and [^18^F]FPyOBn are designed for late-stage radiofluorination of peptides [39]. Both agents were labelled starting from a precursor containing 1,4-diazabicyclo[2.2.2]octane (DABCO) as the leaving group within 10 min reaction at 40 °C for [^18^F]FPyNHS and 80 °C for [^18^F]FPyOBn (Table S1, entries 13, 14 of Supplementary Materials). [^18^F]FPyNHS was labelled with 16.4 ± 3.2% (2% H_2_O) and 5.0 ± 3.4% (5% H_2_O) RCC, respectively (Figure 6). [^18^F]FPyOBn was produced with 97.9 ± 0.2% (*n* = 2) and 95.7 ± 0.6% (*n* = 2) RCC, respectively (Figure 6). [^18^F]FPyZIDE is a prosthetic reagent for different click-labelling reactions [40]. [^18^F]FPyZIDE was produced starting either from a nitro precursor or a trimethylammonium precursor within 10 min reaction at 130 °C in DMSO (Table S1, entries 17–20 of Supplementary Materials). With 2% H_2_O, [^18^F]FPyZIDE was labelled with 12.6 ± 2.4% (NO_2_ precursor) and 60.9 ± 14.1% (NMe_3_^+^ precursor) RCC, respectively. With 5% H_2_O, the RCCs were 1.4 ± 0.9% and 27.1 ± 2.6%, respectively (Figure 6).

## 3. Discussion

In this study, two hydrous F-18 activation methods based on (1) cryptate-mediated (kryptofix) ^18^F-fluorination or (2) using concentrated TBAB solution have been evaluated based on the literature data (Table 1) along with three different anion exchangers. The manual ^18^F-elution tests have shown that the three types of beads gave trapping efficiencies of fluorine-18 higher than 92%. The elution efficiency was around 20% superior for Waters^TM^ and Eichrom beads compared to S*Pure beads. A comparison of elution approaches showed that the K_222_-based method gave better results than the TBAB-based method. The elution efficiency is 20% higher for K_222_ independently from the bead type. An optimal volume of elution of 0.5 mL has been determined, while the first 0.1 mL may be discarded due to the very low amount of radioactivity it contains. The best beads and elution method combination was the QMA-CO_3_ beads from Waters^TM^ eluted with 0.5 mL of a K_222_ solution containing 3% water in acetonitrile. The kryptofix-based elution method was transferred to the iMiDEV^TM^ microfluidic platform, but the elution volume had to be reduced because the reactor content (R2) on the iMiDEV^TM^ cassette cannot exceed 286 µL. Nevertheless, fractioned elution showed that 70% and even 90% of the radioactivity could be eluted within 0.2 and 0.3 mL of PTC solution, respectively. These observations are encouraging for the transfer on the iMiDEV^TM^ microfluidic platform.

On the iMiDEV™ cassettes, reactor 1, dedicated to the trapping and concentration of [^18^F]F^−^ (R1, volume 50 µL), was filled with QMA-CO_3_ beads from Waters^TM^ due to their optimal trapping and elution efficiencies. Only the hydrous K_222_-based method has been evaluated using a large panel of elution volumes from 0.1 to 1 mL and water content from 1 to 5%. Trapping efficiency was almost quantitative, reaching 99% without any loss during the washing and drying steps of the beads. Elution efficiency increased with increasing elution volume and water content. Only half of the radioactivity could be eluted with 0.1 mL of elution solution, whatever the water content, along with a high variance in elution efficiency. This result is in accordance with the manual elution study in which only very low amounts of radioactivity could be eluted using the first 0.1 mL. Moreover, Mallapura et al. discussed in a previous study that the cassette and vials clamping step on the docking plate of the iMiDEV™ platform may conceal small volumes in vials, leading to variable dead volumes affecting the subsequent elution step [41]. With higher volumes (0.15, 0.2 and 1 mL), elution efficiency increased and reached 95 to 100%, with the highest water content (5%). Nevertheless, a volume of 1 mL is not relevant regarding the volume of R2 (286 µL) on the iMiDEV™ cassette. This set of elution efficiency measurements showed that a minimum of 3 bed volumes of chamber R1 (i.e.*,* 0.15 mL) of elution solution containing at least 2% water leads to satisfactory elution efficiencies. This is a suitable volume considering the needed precursor volume (0.15 mL) and reactor R2 volume on the iMiDEV™ cassette. To compare with earlier microelution studies with AEX beads, we have reached a similar elution efficiency using 3–4 bed volumes of 2–5% water-containing kryptofix eluent, what has earlier been reached using 4 bed volumes of fully aqueous basic eluents [34,42].

We focussed then on the evaluation of the radiochemical conversion (measured by radio-TLC) of various precursors representing most of the possible options for nucleophilic radiofluorination. Aliphatic radiofluorination was performed on five precursors displaying different leaving groups, such as a tosylate (precursors of [^18^F]DPA-714, [^18^F]fallypride and [^18^F]F-Me-OTs), a chlorine atom (precursor for [^18^F]LBT-999) or a triflate moiety (precursor for [^18^F]FTAG). Satisfactory to good RCCs were observed for all precursors despite the PTC solution used. Damont et al. [43] described RCC ranging from 50 to 70% for [^18^F]DPA-714, and Gao et al. [44]. reported the best d.c. RCC, reaching 50% for [^18^F]fallypride, which is fully in accordance with our observations. For [^18^F]LBT-999, our RCC was comparable with the n.d.c. radiochemical yields reported by Vala et al. [45]. For the aliphatic radiofluorination, the RCCs tend to slightly decrease when 5% water compared to 2% water in K_222_ solution was used, which is not the case when using 5% water in TBAB_40%_ solutions, leading to the highest [^18^F]DPA-714 and [^18^F]FTAG RCCs. For the preparation of [^18^F]F-Me-OTs, several reports indicated that the presence of water increased the production yield of [^18^F]FCH_2_OTs while limiting the formation of the [^18^F]tosylfluoride by-product [36,37,38]. In batch chemistry, Neal et al. optimised the amount of water to 5% and obtained [^18^F]FCH_2_OTs in 83% d.c. yield. They also observed that conversion was higher using kryptofix rather than TBAB, a difference in PTC performance that we have not observed [38]. Rodnick et al. introduced 13% water in their cyrptate-based solution and obtained the desired intermediate in 38% RCC [36]. The preparation of [^18^F]FCH_2_OTs was also implemented on the Avdion^TM^ microfluidic device. Pascali et al. showed that a significant amount of water (optimised to 20%) was necessary to promote the formation of the desired radioactive intermediate (n.d.c. RCY of 44%) and to avoid the production of [^18^F]FOTs [36]. We observed in our study that adding water (5 to 10%) to the reaction mixture does not hamper the radiofluorination, and our RCCs are also higher than the ones previously reported.

Heteroaromatic radiofluorination was performed on four various precursors of prosthetic reagents. [^18^F]FPyOBn, starting from a quaternary ammonium precursor, was obtained with the highest RCCs, probably due to the strong activation of the pyridine ring provided by the benzyl ester. The content of water did not seem to have any influence on the conversion rate. The activated ester, [^18^F]FPyNHS, was obtained with modest and even low RCC when the content of water in the eluent was increased from 2 to 5%, respectively. Partial hydrolysis of the activated ester due to the presence of water in basic conditions may be the reason for such low RCCs. Richard et al. reported comparable conversion yields for [^18^F]FPyOBn but drastically higher conversions for [^18^F]FPyNHS. Note that reactions were conducted in standard anhydrous conditions at lower temperatures [39]. Finally, the preparation of azide-containing reagent, [^18^F]FPyZIDE, was evaluated starting either from a nitro or a quaternary ammonium precursor. Nitro-precursor gave only low RCCs at 130 °C. In a previous report, Kuhnast et al. [46] showed that the radiofluorination of such a 2-nitro-3-alkoxypyridine analogue gave very high yields (>90%) at 165 °C in a 3 min reaction. In this study, we chose 130 °C as the maximum temperature to be compatible with the future transfer to the iMiDEV^TM^ platform. The quaternary ammonium precursor gave good RCCs when using 2% water in the PTC solution. Again, in this latter case, increasing the amount of water drastically decreased the RCC. Overall, 2% water in K_222_ solution was well tolerated by most of the tested precursors, and the increase of water content to 5% adversely affected the radiochemical conversion rates. When TBAB solution was used as PTC, the presence of water seemed to be better tolerated and gave even better RCCs compared to 5% water in K_222_ solutions.

## 4. Materials and Methods

### 4.1. Chemicals

*N*,*N*-Diethyl-2-(2-(4-(2-tosyloxy-1-ethoxy)phenyl)- 5,7-dimethylpyrazolo[1,5-*a*]pyrimidin-3-yl)acetamide (TsO-DPA-714), *N,N*-Diethyl-2-(2-(4-(2-fluoro-1-ethoxy)phenyl)- 5,7-dimethylpyrazolo[1,5-a]pyrimidin-3-yl)acetamide (DPA-714), were purchased from Pharmasynth (Estonia). (S)-2,3-Dimethoxy-5-[3-[[(4-methylphenyl)-sulfonyl]oxy]propyl]-N-[[1-(2-propenyl)-2-pyrrolidinyl]methyl]benzamide (TsO-fallypride), (S)-5-(3-Fluoropropyl)-2,3-dimethoxy-*N*-[[(2S)-1-(2-propenyl)-2-pyrrolidinyl]methyl]benzamide (fallypride), mannose triflate plus (ultra-pure) precursor for [^18^F]FDG and bis(tosyloxy)methane precursor for [^18^F]F-Choline were purchased from ABX (Germany). LBT-999 (8-((*E*)-4-fluoro-but-2-enyl)-3-beta-*p*-tolyl-8-aza-bicyclo[3.2.1]octane-2-beta-carboxylicacid methyl ester) and its chloro-precursor (8-((*E*)-4-chloro-but-2-enyl)-3-beta-*p*-tolyl-8-aza-bicyclo[3.2.1]octane-2-beta-carboxylicacid methyl ester) were obtained from ERAS Labo (France). 3-(2-(2-(2-azidoethoxy)ethoxy)ethoxy)-2-nitropyridine (nitro-precursor for [^18^F]FPyZIDE), 3-(2-(2-(2-azidoethoxy)ethoxy)ethoxy)-*N,N,N*-trimethylpyridin-2-aminium triflate (trimethylammonium-precursor for [^18^F]FPyZIDE), 3-(2-(2-(2-azidoethoxy)ethoxy)ethoxy)-2-fluoropyridine ([^18^F]FPyZIDE reference), 3-((2,5-dioxo-1-pyrrolidinyl)-carbonyl)pyridin-6-(4-aza-1-azoniabicyclo[2.2.2]octane) trifluoromethanesulfonate (precursor for [^18^F]FPyNHS), 2,5-dioxopyrrolidin-1-yl 6-fluoronicotinate ([^18^F]FPyNHS reference), 1-(4-(phenoxycarbonyl)phenyl)-1,4-diazabicyclo[2.2.2]octan-1-ium triflate (precursor for [^18^F]FPyOBn) and phenyl 4-fluorobenzoate ([^18^F]FPyOBn reference) were synthetised at SHFJ as previously described [39,40].

Anhydrous acetonitrile (˃99.8%), K_2_CO_3_ 99.99%, kryptofix^®^ K_2.2.2_ (4,7,13,16,21,24-hexaoxa-1,10-diazabicyclo-[8.8.8]-hexacosane), ammonium acetate, trifluoroacetic acid (TFA) and dimethyl sulfoxide (DMSO) were purchased from Sigma Aldrich (Steinheim, Germany). Acetonitrile (HPLC grade) was purchased from CARLO ERBA Reagents (Val-de-Reuil, France). TBA.HCO_3_ aqueous solution (40% stock solution, TBAB) was purchased from FUTURECHEM Co., Ltd. (Seoul, Republic of Korea).

Sterile water for injections (WFI) Macoflex N from Macopharmas; 0.9% Sodium chloride (NaCl) solution for injections was purchased from Baxter^®^. Pure H_2_O (18.2 MΩ) was produced with a purification system (ARIUM^®^ MINI, Göttingen, Germany).

### 4.2. Eluent Preparation

#### 4.2.1. Preparation of K_222_/K_2_CO_3_/CH_3_CN/H_2_O Solutions

Preparation of different stock solutions of K_222_/K_2_CO_3_/acetonitrile/water was performed using amounts and volumes described in Table 2.

Each solution was freshly prepared and ultrasonicated until the complete homogenisation of the eluent prior to use. Water content was checked using the Karl Fisher method.

#### 4.2.2. Preparation of 5% H_2_O TBAB_40%_ Solution

In a vial, 50 µL of concentrated TBAB (40%) was added to 1 mL of acetonitrile. The resulting freshly prepared solution was vortexed to ensure thorough mixing.

##### 4.3. [^18^F]fluoride Production

Aqueous [^18^F]fluoride was produced via the ^18^O(p,n)^18^F nuclear reaction from ^18^O-enriched H_2_O on a PET Trace cyclotron from General Electric (Windsor, CT, USA) at the CURIUM^TM^ facility (Nancy, France) and a Cyclone-18/9 Cyclotron (IBA) at the SHFJ (Orsay, France).

### 4.4. General Manual [^18^F]fluoride Elution Method

Quaternary methyl ammonium carbonate (QMA-CO_3_) anion-exchange (AEX) SPE cartridges were purchased from Waters^TM^ (Sep-Pak^®^ Accell Plus QMA Carbonate, 37–55 µm particle size, Guyancourt, France), Eichrom (QMA-S-BC, 55 µm particle size, Lisle, IL, USA) and S*Pure (Maxi-Clean QMA Carbonate, 50 µm particle size, Singapore), and the packing material was reduced to 25 mg to mimic the mass available in the R1 reactor of the microfluidic cassette iMiDEV^TM^. Aqueous [^18^F]fluoride (0.5–0.75 mL) was passed through an anion-exchange cartridge (without preconditioning) followed by 5 mL air, and the activity of the cartridge was determined with a dose calibrator (CRC^®^-25R, Capintec, Inc., Florham Park, NJ, USA). The cartridge was rinsed with 4 mL anhydrous acetonitrile to eliminate the residual water and dried with 15 mL air. [^18^F]fluoride was fractionally eluted from the reverse direction using two different elution solutions (3% K_222_/K_2_CO_3_/CH_3_CN/H_2_O or 5% water TBAB_40%_) followed by 5 mL of air per fraction. Elution fractions were 0.1 mL, and the total elution volume was 0.5 mL. The activity of the eluate fraction and AEX cartridge (residual [^18^F]fluoride) was determined with a Capintec dose calibrator. Activity data was used to calculate % fluoride recovery.

### 4.5. General [^18^F]fluoride Elution Method Using iMiDEV^TM^

The microfluidic-based platform iMiDEV™ (PMB-Alcen, Peynier, France) uses disposable microfluidic cassettes (PMB-Alcen, France) for radiotracer production. Characteristics of the device and the microfluidic cassettes have been described earlier [47]. In brief, the cassettes have four independent chambers, where chamber R1 is dedicated to radionuclide concentration and chamber R2 is dedicated to the labelling reaction. R1 has a volume of 50 µL (dimensions W 5.5 mm, L 10 mm, H 1 mm), and R2 has a volume of 286 µL (dimensions W 12 mm, L 36 mm, H 1 mm). In this work, to study elution in microfluidic conditions, we used cassettes where chamber R1 was filled with approximately 25 mg QMA-CO_3_ anion-exchange beads (Waters™). R1 is filled during the manufacturing of the cassettes, as previously described [48]. Briefly, filling is done through a hole on top of chamber R1; the initial filling is based on gravity, and to make the bead distribution around the chamber even, compressed air is pushed through, and ultrasonic vibrations are used.

Aqueous [^18^F]fluoride (2 mL) was passed through the nonpreconditioned QMA beads, followed by a stream of He for drying for 2 min at 1.2 bar. Beads were washed with CH_3_CN (4 mL in vial C/D), followed by a stream of He for drying for 3 min at 2 bar. [^18^F]fluoride was then eluted with K_222_/K_2_CO_3_/CH_3_CN eluent solution (0.1 to 1 mL in vial A/B), followed by a stream of He for 0.5 min at 0.2 bar. Elution efficiency (EE) and activity loss during the washing step were calculated using the radioactivity sensor data. A radioactivity sensor is placed at the outlet of the R1 reactor. Cassette architecture and the elution pathways are presented in the supporting information (Figures S1 and S2 of Supplementary Materials).

### 4.6. General Hydrous [^18^F]fluoride Labelling Method

Molecules selected to study manual labelling reaction were [^18^F]FTAG (intermediate in [^18^F]FDG synthesis), [^18^F]F-Me-OTs (intermediate in [^18^F]fluoromethylcholine ([^18^F]FCH) synthesis), [^18^F]DPA-714, [^18^F]fallypride, [^18^F]LBT-999 and prosthetic reagents [^18^F]FPyNHS, [^18^F]FPyZIDE and [^18^F]FPyOBn. Labelling reactions were performed manually. A fraction of ^18^F-eluate (150 µL, 50–200 MBq) was mixed with the precursor (2–20 µmol) dissolved in 150 µL of the polar aprotic solvent (CH_3_CN or DMSO). Reaction conditions for each precursor are detailed in Table S1 of Supplementary Materials. The corresponding mixture was heated according to commonly used labelling conditions. At the end of the labelling, the mixture was cooled down, and an aliquot of the crude reaction solution was used for the quality control tests. The radiochemical conversion is determined by radio-TLC analysis of a small aliquot from a reaction solution and identity using radio-HPLC analysis (Table S2 of Supplementary Materials). Each labelling was done in triplicate.

### 4.7. Quality Control Analysis

Radio-TLCs were performed either with a Mini GITA TLC scanner controlled by GinaX 10.4.5 software (Elysia S.A., Angleur, Belgium) or a Mini-Scan and Flow-Count radioactive detection system (Bioscan) and Chromeleon software 7.2.10.ES (Thermo Fischer, Courtaboeuf, France). An aliquot of the reaction mixture (5 µL) was analysed by radio-TLC on a silica-gel-coated aluminium plate. Elution conditions and Rf are detailed in the table below (Table 3).

## 5. Conclusions

In this study, we have optimised several conditions to allow the implementation of hydrous radiofluorination approaches at the microfluidic scale on the iMiDEV™ platform. We first tested manually different anion exchangers to trap fluorine-18 and two PTC solutions to release it prior to radiofluorination. We observed that QMA-CO_3_ beads from Waters^TM^ gave the best results combined with a cryptate-based PTC solution for the recovery of fluorine-18. Fractioned elution showed that 90% of radioactivity could be released within 0.3 mL, which is compatible with the iMiDEV^TM^ microfluidic platform. These results were confirmed on the microfluidic platform with even better elution efficiencies using only 0.15 to 0.2 mL of PTC solution. We then evaluated the hydrous radiofluorination of seven different aliphatic and heteroaromatic precursors with different leaving groups. Two standard PTC solutions containing various amounts of water were tested. Good to high radiochemical conversions were observed for the aliphatic radiofluorination whatever the PTC solution used. For the aromatic radiofluorination, results are more contrasted, probably due to the presence or absence of an electron-withdrawing group on the pyridine ring and to the temperature that we have limited to 130 °C. Altogether, the results show that most of the tested precursors tolerate the presence of water, but an increase in water led to a decrease in the radiofluorination radiochemical conversions. This study opens access to a variety of radiopharmaceuticals using hydrous radiofluorination approaches, which could be integrated into new emerging microfluidic platforms.

## Figures and Tables

**Table 1:** Overview of various ^18^F-radiolabelling methods via nucleophilic substitution in hydrous conditions.

Entry	Method	Tracer(s)	Conditions	Advantages/Drawbacks	Reference
1	“^18^F-radiofluorination on SPE cartridge”	6-[^18^F]FPy-TFP; 6-[^18^F]SFPy; [^18^F]FFNP; [^18^F]FDHT	^18^F-Elution of triflate precursor in organic solvent through PS-HCO_3_	Simplicity of processing Suitable for base and temperature-sensitive starting materials High yields Limited applicability to multiple radiopharmaceuticals	Basuli et al., 2019, 2020 [5,6]
2	^18^F-radiofluorination on modified polymers	[^18^F]FLT, [^18^F]FDG and Silicon-based prosthetic groups	^18^F-Elution of precursor in organic solvent through modified solid support	Simplicity of processing Column reusability Variability in RCY Column accessibility and packing effects A large amount of precursors required	Mathiessen et al., 2013 [7]; Aerts et al., 2010 [8]; Balentova et al., 2011 [9]
3	^18^F-radiofluorination using strong bases	[^18^F]FDG and several other aliphatic and aromatic substrates	^18^F-Elution and activation with phosphazene bases	High yields Limited application because of strong basicity No standard test for residual phosphazenes quantification	Lemaire et al., 2010 [10]; Mathiessen et al., 2011 [11]
4	Ionic liquids	Halo- and mesyloxyalkanes, [^18^F]FDG	Labelling in the presence of various ionic liquids ([bmim][OTf], BMI)	Shorter synthesis time and simplified reaction procedure Limited substrate scope No standard test for residual ionic liquid quantification	Kim et al., 2003, 2004 [12,13]
5	Transition metal mediated/catalyzed radiofluorination	Wide range of substrates	^18^F-Elution with phase transfer catalyst (PTC) or organic base and mixing with precursor in the presence of transition metal containing catalyst	High radiofluorination efficiency Accessibility and versatility of suitable precursors Metal dosage required for QC Complicated automation and scale-up	Sergeev et al., 2015 [14]; Mossine et al., 2017 [15]; Zischler et al., 2017 [16]; Scroggie et al., 2021 [17]; Liu et al., 2022 [18]; Klenner et al., 2017 [19]
6	Cryptate-mediated ^18^F-fluorination	Wide range of substrates	^18^F-Elution with inorganic base and kryptofix 2.2.2 followed by drying the cartridge with acetonitrile and labelling	Applicability to versatile commercial precursors Standard QC procedure	Stewart et al., 2015 [20]; Lindner et al., 2016 [21]; Kniess et al., 2017 [22]; Wessmann et al., 2017 [23]; Kwon et al., 2018 [24]
7	Tetraalkylammonium salts (“non-anhydrous, minimally basic (NAMB) approach”)	Wide range of substrates	^18^F-Elution with tetraalkylammonium salt followed by drying the cartridge with acetonitrile and labelling	Applicability to versatile commercial precursors Standard QC procedure	Inkster et al., 2020 [25] Seok Moon et al., 2010 [26] Brichard et al., 2014 [27]; Kwon et al., 2018 [28]; Wenzel et al., 2019 [29]
8	Sulfonyl-^18^F	A wide range of substrates and sulfonyl fluoride-containing molecules	Production of sulfonyl-[^18^F]fluoride followed by distillation or SPE purification prior to radiolabelling	Applicability to versatile commercial precursors Complicated automation	Zhou et al., 2023 [30]; Pees et al., 2018 [31]; Zhang et al., 2019 [32]

**Table 2:** Preparation details for different K_222_/K_2_CO_3_/CH_3_CN/H_2_O solutions.

Eluent	Amount of K_222_	Addition of Aq. K_2_CO_3_ Solution (Corresponding to 150 µmol)	Volume of CH_3_CN
K_222_ with 5% H_2_O	113 mg (300 µmol)	250 µL of 0.6 M	5 mL
K_222_ with 3% H_2_O		150 µL of 1 M	
K_222_ with 2% H_2_O		100 µL of 1.5 M	
K_222_ with 1% H_2_O		50 µL of 3 M	

**Table 3:** Radio-TLC analyses of radiofluorinated compounds.

Radiotracer	Eluant Mixture (*v*:*v*)	Rf
[^18^F]f^−^	MeCN:H_2_O (95:5)	0
[^18^F]f^−^	EtOAc (100)	0
[^18^F]f^−^	Hexanes:EtOAc (80:20)	0
[^18^F]FTAG	MeCN:H_2_O (95:5)	0.47
[^18^F]F-Me-OTs	Hexanes:EtOAc (80:20)	0.45
[^18^F]Tosyl fluoride	Hexanes:EtOAc (80:20)	0.57
[^18^F]DPA-714	MeCN:H_2_O (95:5)	0.9
[^18^F]DPA-714	EtOAc (100)	0.4
[^18^F]fallypride	EtOAc (100)	0.4
[^18^F]LBT-999	EtOAc (100)	0.4
[^18^F]FPy-NHS	EtOAc (100)	1
[^18^F]FPyZIDE	EtOAc (100)	0.9
[^18^F]FPyOBn	EtOAc (100)	1

## Data Availability

Data are contained within the article and Supplementary Materials.

## Supplementary Materials

The following supporting information can be downloaded at https://www.mdpi.com/article/10.3390/molecules29010147/s1; Figure S1: Microfluidic cassette; Figure S2: Elution pathways inside microfluidic cassette; Table S1: Nucleophilic ^18^F-fluorination conditions for each labelled tracer/prosthetic agent. Details of ra-dio-HPLC; Table S2: HPLC conditions; Table S3: Comparison of the mass of QMA-CO_3_ beads be-tween different suppliers; Table S4: Results from manual elution using kryptofix-based method; Table S5: Results from manual elution using TBA-based method; Table S6: Results from microfluidic elution using kryptofix-based method.
