# Supplementary material for: [18F]fluoride Activation and 18F-Labelling in Hydrous Conditions—Towards a Microfluidic Synthesis of PET Radiopharmaceuticals
Source: Molecules. 2023 Dec 26;29(1):147. doi: 10.3390/molecules29010147 (PMC10779751; doi:10.3390/molecules29010147)
Supplement: Supplementary file 1 [file molecules-29-00147-s001.zip › molecules-2713474-supplementary.pdf]

SUPPLEMENTARY INFORMATION
[^{18}F]Fluoride activation and ^{18}F-labelling in hydrous conditions – towards a microfluidic synthesis of PET radiopharmaceuticals

Olga Ovdiichuk,*[1] Salla Lahdenpohja,[2] Quentin Béen,[1] Laurent Tanguy,[3] Bertrand Kuhnast,[2] Charlotte Collet-Defossez [1,4]

[a] Nancyclotep, Molecular Imaging platform, 54500 Vandoeuvre-les-Nancy, France
[b] Université Paris Saclay, CEA Inserm, CNRS, BioMaps, 91401 Orsay, France
[c] PMB-Alcen, 13790 Peynier, France
[d] Université de Lorraine, Inserm, IADI, F-54000 Nancy, France
*****Correspondence: oovdiichuk@nancyclotep.com (OO)

Content

Figure S1. Microfluidic cassette.
Figure S2. Elution pathways inside microfluidic cassette.
Table S1. Nucleophilic ^{18}F-fluorination conditions for each labelled tracer/prosthetic agent.
Details of radio-HPLC.
Table S2. HPLC conditions.
Table S3. Comparison of the mass of QMA-CO_3 beads between different suppliers.
Table S4. Results from manual elution using kryptofix-based method.
Table S5. Results from manual elution using TBA-based method.
Table S6. Results from microfluidic elution using kryptofix-based method.

Figure S1. Picture of iMiDEV™ microfluidic cassette

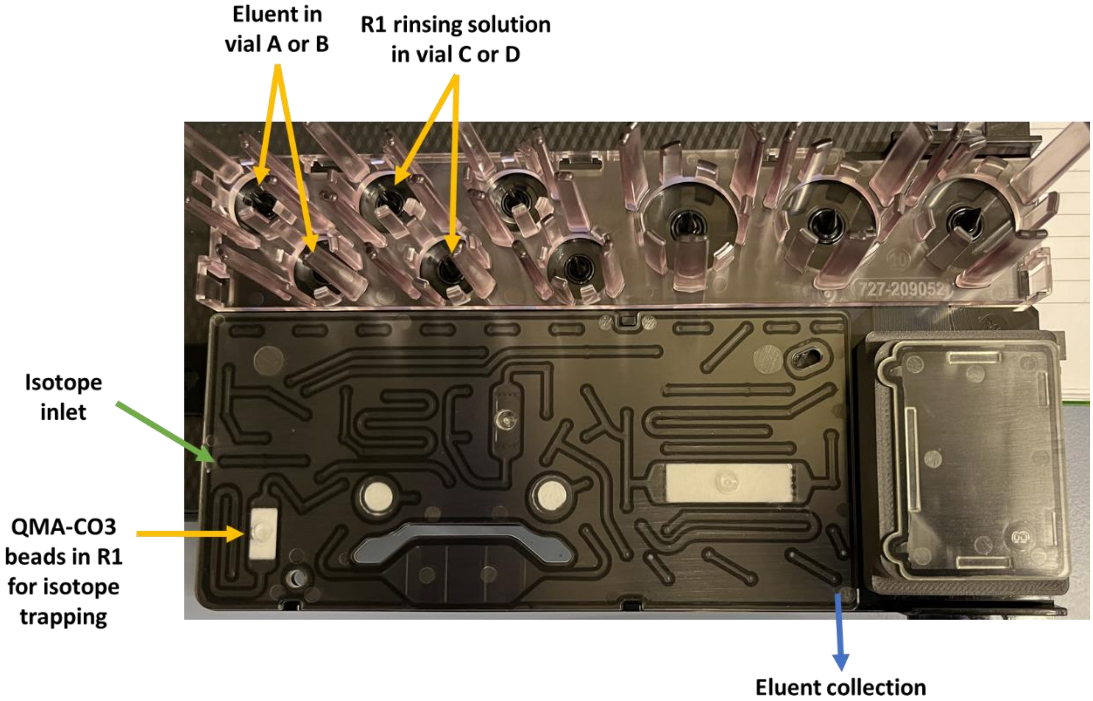

Figure S2. Cassette architecture with liquid and gas pathways marked in green, orange and yellow. A) Trapping [^{18}F]fluoride on QMA-CO_3 (Waters®) beads in R1; B) rinsing the QMA-CO_3 beads with MeCN from C (green) or alternatively from D (orange); C) elution [^{18}F]fluoride from R1 with eluent in A (green) or alternatively in B (orange).

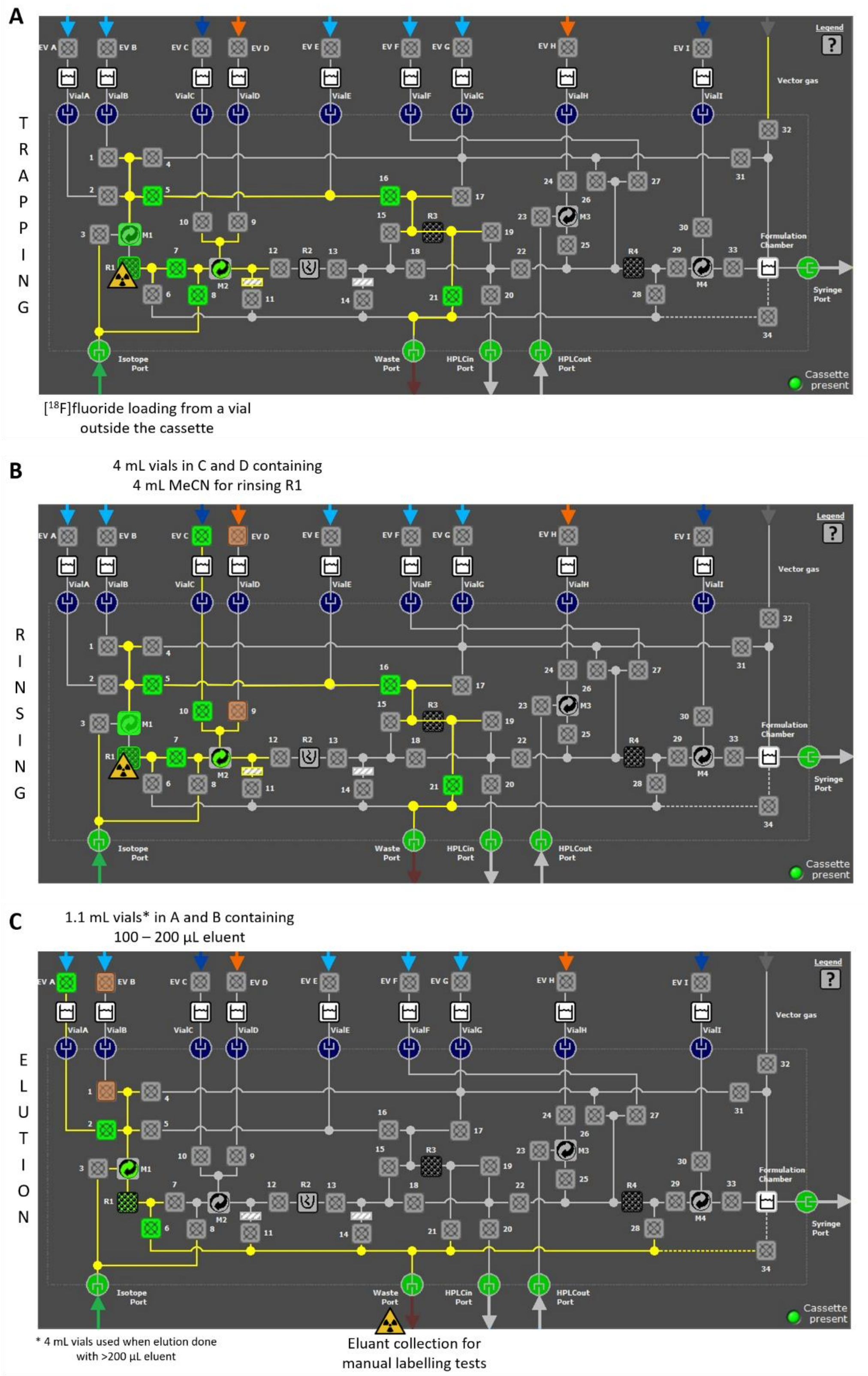

Table S1. Results of the azeotropic drying free nucleophilic ^{18}F-fluorination (n = 3).

Entry	Product	Precursor quantity	Activating agent (150 µL)	Precursor Solvent (150 µL)	Labelling conditions	RCC, %
1	[^{18}F]FTAG	5 mg (10.4 µmol)	3% H_2O K_{222}/K_2CO_3 in CH_3CN	100% CH_3CN	95 °C 5 min	82.2±5.8
2	[^{18}F]FTAG	5 mg (10.4 µmol)	5% H_2O $TBAB_{40\%}$ in CH_3CN	100% CH_3CN	95 °C 10 min	89.9±2.4
3	[^{18}F]F-Me-OTs	7 mg (19.6 µmol)	3% H_2O K_{222}/K_2CO_3 in CH_3CN	9/1 CH_3CN/ H_2O	120 °C 10 min	63.9±4.3
4	[^{18}F]F-Me-OTs	7 mg (19.6 µmol)	3% H_2O K_{222}/K_2CO_3 in CH_3CN	8/2 CH_3CN/ H_2O	120 °C 10 min	49.2±17.8
5	[^{18}F]F-Me-OTs	7 mg (19.6 µmol)	5% H_2O $TBAB_{40\%}$ in CH_3CN	8/2 CH_3CN/ H_2O	120 °C 10 min	49.7±0.9
6	[^{18}F]DPA-714 (OTs prec)	1.5 mg (2.7 µmol)	2% H_2O K_{222}/K_2CO_3 in CH_3CN	100% DMSO	130°C 10 min	52.5±10.1
7	[^{18}F]DPA-714 (OTs prec)	1.5 mg (2.7 µmol)	5% H_2O K_{222}/K_2CO_3 in CH_3CN	100% DMSO	130°C 10 min	41.2±9.0
8	[^{18}F]DPA-714 (OTs prec)	1.2 mg (2.2 µmol)	5% H_2O $TBAB_{40\%}$ in CH_3CN	100% DMSO	130 °C 10 min	66.0±10%
9	[^{18}F]Fallypride (OTs prec)	1.0 mg (1.9 µmol)	2% H_2O K_{222}/K_2CO_3 in CH_3CN	100% DMSO	130 °C 10 min	41.6±9.8
10	[^{18}F]Fallypride (OTs prec)	1.0 mg (1.9 µmol)	5% H_2O K_{222}/K_2CO_3 in CH_3CN	100% DMSO	130 °C 10 min	18.7±1.4 (n=2)
11	[^{18}F]LBT-999 (Cl prec)	1.5 mg (4.3 µmol)	2% H_2O K_{222}/K_2CO_3 in CH_3CN	100% DMSO	130 °C 10 min	39.5±1.7
12	[^{18}F]LBT-999 (Cl prec)	1.5 mg (4.3 µmol)	5% H_2O K_{222}/K_2CO_3 in CH_3CN	100% DMSO	130 °C 10 min	29.8±1.4
13	[^{18}F]FPyNHS (DABCO prec)	2.4 mg (4.9 µmol)	2% H_2O K_{222}/K_2CO_3 in CH_3CN	100% DMSO	40 °C 10 min	16.4±3.2
14	[^{18}F]FPyNHS (DABCO prec)	2.5 mg (5.2 µmol)	5% H_2O K_{222}/K_2CO_3 in CH_3CN	100% DMSO	40 °C 10 min	5.0±3.4
15	[^{18}F]FPyOBn (DABCO prec)	1.3 mg (2.7 µmol)	2% H_2O K_{222}/K_2CO_3 in CH_3CN	100% DMSO	80 °C 10 min	97.9±0.2 (n=2)
16	[^{18}F]FPyOBn (DABCO prec)	1.2 mg (2.5 µmol)	5% H_2O K_{222}/K_2CO_3 in CH_3CN	100% DMSO	80 °C 10 min	95.7±0.6 (n=2)
17	[^{18}F]FPyZIDE (Me_3N^+ prec)	1.3 mg (2.8 µmol)	2% H_2O K_{222}/K_2CO_3 in CH_3CN	100% DMSO	130 °C 10 min	60.9±14.1
18	[^{18}F]FPyZIDE (Me_3N^+ prec)	1.4 mg (3.0 µmol)	5% H_2O K_{222}/K_2CO_3 in CH_3CN	100% DMSO	130 °C 10 min	27.1±2.6
19	[^{18}F]FPyZIDE (NO_2 prec)	1.2 mg (4.0 µmol)	2% H_2O K_{222}/K_2CO_3 in CH_3CN	100% DMSO	130 °C 10 min	12.6±2.4
20	[^{18}F]FPyZIDE (NO_2 prec)	1.2 mg (4.0 µmol)	5% H_2O K_{222}/K_2CO_3 in CH_3CN	100% DMSO	130 °C 10 min	1.4±0.9

Details of radio-HPLC analyses:

HPLCs were performed on:

[HPLC A]: Alliance e2695 system equipped with a 2998 PDA detector and Herm LB500 equipped with NaI detector (Berthold) controlled by the Empower Software (Waters), Zorbax SB-C18 5 µm 4.6 × 250 mm analytical column (Agilent).

[HPLC B]: Alliance e2695 system equipped with a 2998 PDA detector and Herm LB500 equipped with NaI detector (Berthold) controlled by the Empower Software (Waters), Symmetry-M® C-18 5µm 150 × 4.6 mm analytical column (Waters).

[HPLC C]: Alliance 2690 system equipped with a 996 PDA detector (Waters) and a LB509 radioactivity detector (Berthold) controlled by the Empower Software (Waters), Symmetry-M® C-18 5µm 50 x 4.6 mm analytical column (Waters).

Elutions were performed using the following solvents:

S1: water containing 0.1% TFA

S2: acetonitrile containing 0.1% TFA

S3: water containing Low-UV PIC® B7 reagent (20 mL for 1000 mL) (Waters)

S4: water:acetonitrile 30:70 (v:v) containing Low-UV PIC® B7 reagent (20 mL for 1000 mL) (Waters)

Solvent mixtures, flow rates, λ detection and retention times of radiotracers are summarized in Table S2.

Table S2: HPLC conditions.

Radiotracer	HPLC system	Eluant mixture (v:v)	Flow rate (mL/min)	λ (nm)	Radio-HPLC Rt (min)
[^{18}F]F-Me-OTs	A	S1:S2 (55:45)	1	254	9.6
[^{18}F]Tosyl fluoride	A	S1:S2 (55:45)	1	254	13.3
[^{18}F]DPA-714	B	S1:S2 (55:45)	1	263	3.9
[^{18}F]DPA-714	C	S3:S4 (40:60)	2	254	2.1
[^{18}F]Fallypride	C	S3:S4 (60:40)	2.5	220	1.6
[^{18}F]LBT-999	C	S3:S4 (55:45)	2	220	2.2
[^{18}F]FPyNHS	C	S3:S4 (75:25)	2	260	1.8
[^{18}F]FPyZIDE	C	S3:S4 (60:40)	2	267	2.6
[^{18}F]FPyOBn	C	S3:S4 (35:65)	2	254	2.7

Table S3. Comparison of the mass of anion exchange beads between different suppliers

Mass\Supplier	Waters Sep-Pak Accell Plus QMA Carbonate Light Cartridge	Eichrom QMA-S-BC, (QMA-CO_3)	Maxi-Clean S-Pure QMA-CO_3
Mass declared by supplier	130 mg	125 mg	125 mg
Mass measured (n = 3)	108±1 mg	118.7±0.4 mg	54.4±1.9 mg

Table S4. Results of the elution efficiencies (EE) obtained in manual [18F]fluoride fractionated elution tests using kryptofix-based method (n = 3) and 3 different QMA-CO_3 types of beads (25 mg/cartridge)

Entry	Volume of eluent (K_{222}/K_2CO_3/MeCN, 3% H_2O)	EE average (Waters)	EE average (Eichrom)	EE average (S*Pure)
1	0.1 mL	2.5±1.2%	7.3±6.8%	0.3±0.3%
2	0.2 mL	34.4±2.6%	41.8±6.3%	4.7±3.6%
3	0.3 mL	67.5±4.3%	73.4±9.4%	20.1±10.7%
4	0.4 mL	86.3±3.1%	90.0±5.3%	43.1±12.7%
5	0.5 mL	94.1±1.4%	96.4±2.9%	68.3±7.6%

Table S5. Results of the elution efficiencies (EE) obtained in manual [18F]fluoride fractionated elution tests using tetrabutylammonium-based method (n = 3) and 3 different QMA-CO_3 types of beads (25 mg/cartridge)

Entry	Volume of eluent (TBAB 5%)	EE average (Waters)	EE average (Eichrom)	EE average (S*Pure)
1	0.1 mL	0.6±0.5%	0.1±0.1%	0.1±0.2%
2	0.2 mL	16.1±4.7%	10.9±4.6%	3.7±2.6%
3	0.3 mL	40.0±5.4%	36.9±9.8%	14.0±3.5%
4	0.4 mL	60.5±7.5%	55.5±9.8%	30.0±3.6%
5	0.5 mL	73.8±3.4%	70.8±7.4%	47.0±2.3%

Table S6. Results of the [18F]fluoride EE using kryptofix-based elution method (n ≥ 3) obtained in microfluidic cassette using reactor R1 filled with approximately 25 mg of QMA-CO_3 beads from Waters®.

Entry	Volume of eluent (K_{222}/K_2CO_3/CH_3CN eluent)	EE with 1% H_2O	EE with 2% H_2O	EE with 3% H_2O	EE with 5% H_2O
1	0.1 mL	51.4±14.6%	43.3±26.6	46.7±11.2%	55.9±26.8%
2	0.15 mL	69.4±5.4%	82.2±9.4%	90.6±2.5%	94.1±1.9%
3	0.2 mL	70.0±7.4%	86.9±2.9%	92.5±3.9%	93.1±4.5%
4	1 mL	90.8±1.2%	97.6±0.9%	98.2±0.6%	98.7±0.9%
